# A qualitative evaluation of the impact of a Good Life Club on people living with dementia and care partners

**DOI:** 10.1177/1471301221998897

**Published:** 2021-03-20

**Authors:** Lydia Morris, Anthea Innes, Sarah Smith, Jack Wilson, Sophie Bushell, Megan Wyatt

**Affiliations:** School of Health and Society, Institute of Dementia, 5292 University of Salford, UK

**Keywords:** dementia cafe, co-production, care partners, caregivers, psychosocial intervention, gardening

## Abstract

**Background:**

Research suggests there is a lack of post-diagnostic support to enable people living with dementia to fulfil social and active lives throughout their dementia journey. Gardening has been found to have many benefits for people living with dementia. Although such research is important, most research frames people with dementia as passive recipients of stimulation. Research into the impact of a community-based gardening group, where people living with dementia are active in the development of an outdoor space, is underdeveloped. Knowledge about the impact of participating in such groups is also sparse. The Good Life Club (GLC) was co-developed and evaluated to respond to these gaps.

**Objectives:**

The primary aim of this article is to present the findings regarding the impact of attending the GLC on the self-reported well-being for people living with dementia and care partners.

**Methods:**

Qualitative data were collected via 22 semi-structured interviews. Fourteen interviews were conducted before the GLC and eight after the GLC. Thematic analysis was used to analyse data. Dementia Care Mapping data were collected to supplement the interview data.

**Findings:**

Four key themes were identified. The first was that participants considered having active participation in social life to be a key aspect of living a good life. The second was that the way that the GLC was set up and delivered gave the participants ownership of the GLC and within this they felt able to contribute. The third was the importance of social connectedness and peer support to the well-being of both people living with dementia and care partners. Fourth, positive mood and well-being was directly experienced through gardening.

**Conclusions:**

The combination of long-term investment of time and energy to the GLC, ongoing friendships and in-session autonomy act as key ingredients in creating a group that is relaxed, full of humour and highly valued.

Alongside the physical changes associated with the living with dementia, receiving this diagnosis can lead to feelings of anxiety, anger and fear (Bamford et al., 2004). It is common for a person to lose their self-esteem, sense of purpose and confidence in a way referred to as successive losses (Doka, 2010).

Research suggests there is a lack of post-diagnostic support to enable people living with dementia to fulfil social and active lives throughout their dementia journey (Social Care Institute for Excellence, 2015). It is imperative that new initiatives are developed to improve the well-being of those living with dementia (Moniz-Cook et al., 2011). Gardening has many benefits for people living with dementia including improving well-being and reducing disruptive behaviours, whilst also reducing the use of psychotropic drugs and decreasing the risk of falls and incidents (Gonzalez & Kirkevold, 2014; Lu et al., 2020). Noone and Jenkins (2018) articulate that in addition to the reported positive benefits for people living with dementia of being outdoors, gardening provides people with a sense of identity and purpose and facilitates new social relationships that are based on common interests rather than being focussed on a shared diagnosis. Gardening can support sustained well-being for people living with dementia beyond the direct horticultural intervention experience (Hall et al., 2018).

People living with dementia and care partners were core to the consultation process informing the development of a Dementia Hub and garden within the campus of the authors’ institution. Their contributions to this process influenced the overall design and ensuing activities. Within the consultation, the garden space at the front of the Dementia Hub building proved to be of great importance to the people living with dementia and care partners. Specific design features for the outdoor space were considered and implemented. The idea of a ‘Good Life Club (GLC)’ emerged directly from this consultation, and the term was decided by the group members.

The notion of a ‘good life’ is imperative for people living with dementia and has been promoted by policy makers and practitioners (Department of Health & Social Care, 2020). The GLC aimed to address some of the difficulties experienced by those living with or caring for someone with dementia. Although the current research is important in addressing some of the issues associated with dementia, the majority of publications frame people with the condition as passive recipients of stimulation (Noone et al., 2017). Studies with a focus on garden therapy for people with dementia have generally taken place in long-stay care settings (Jarrott & Gigliotti, 2010). Research into the impact of a community-based gardening group, where people living with dementia are active in the development of an outdoor space, is underdeveloped. Knowledge about the impact of actively participating in such groups is also sparse.

The primary aim of this article is to present the findings of the impact of attending the GLC on the self-reported well-being for people living with dementia and care partners. Lessons for others interested in a similar approach are shared.

## Methods

Data were collected before, during and after the intervention. Methods included semi-structured interviews with individuals and care dyads conducted before and after the evaluation of group sessions and structured observations using Dementia Care Mapping (DCM) (Bradford Dementia Group, 2005).

### Participants and procedure

Ethical approval for the study was given at all stages by.

Participants were people living with dementia, their care partners (the University of Salford Ethics committee [HSR1718-062]) and former care partners. In October 2019, 14 participants consented to take part in the evaluation; all participants were able to give informed consent, and the team obtained this by following Dewing (2007) process consent method. Eleven were female and three were male, and the age range for participants was 50–87 years (average age 68.5 years). All participants who expressed an interest in participating were interviewed. Of the 14 participants that were interviewed, four were living with dementia (two women and two men), four were current care partners (three women and one man) and six were former care partners (all women). All four people living with dementia participated as part of a dyad, either with their spouse or other family member.

In March 2020, the UK Government announced ‘lockdown’ guidelines, advising older people, and those at increased risk to shield themselves from COVID-19 (Gov.UK, 2020). In line with these guidelines, the remaining planned eight sessions of the GLC were cancelled. Ethics approval to conduct follow-up interviews virtually was obtained. Eight participants agreed to a telephone or video conferencing interview. Six participants from baseline did not participate in follow-up interviews for the following reasons: two participants (both former care partners) declined to be interviewed, two participants (one person living with dementia and her daughter) had withdrawn from the evaluation after the Christmas break, one participant had sadly passed away (living with dementia) and their carer declined further involvement.

### Interview schedule and data collection

A semi-structured interview schedule was used to conduct interviews in person, over the phone and via video call. Interviews were conducted before the GLC sessions started and then at follow-up (conducted three and a half months after the final session). The length of the interviews ranged between around 30 and 60 minutes. All interviews were digitally recorded and transcribed verbatim. In addition to the questions on the interview schedule, the interviewer asked prompt questions that were integral to the interview schedule. These were designed to encourage participants to elaborate on comments and to obtain a deeper understanding of their experience of the GLC. As this was an exploratory study, convenience sampling was used.

The impact of the GLC was directly observed using DCM during each session, with a particular focus on the mood and well-being of participants. DCM is an observation tool used to evaluate in-the-moment experiences from the perspective of the person living with dementia. By recording individuals’ mood and engagement (ME) at regular intervals (every 5 minutes) throughout the session, using a predefined coding framework, the tool enabled a moment-by-moment examination of the experiences of people living with dementia (Brooker & Surr, 2006).

### Dementia care mapping and unstructured observations

The real-time impact of the GLC was directly observed during each session with a particular focus on the mood and well-being of participants using DCM. DCM is an observation tool used to evaluate the ‘in-the-moment’ experiences from the perspective of the person living with dementia with psychological needs, in particular our focus was using the personal enhancer and detractor coding frame (Bradford Dementia Group, 2005).

### Analytical strategy and procedure

Analysis followed Braun and Clarke (2006) six-phase approach to thematic analysis (see Table 1).

**Table 1.** Braun and Clarke’s (2006) six-phase approach to thematic analysis, including detail of how this was implemented and by whom.

Phases	Application of the phases within this study
Becoming familiar with the data	MW, JW and LM conducted the interviews. Transcripts were repeatedly read.
Generating initial codes	MW and JW coded the data in a systematic fashion across the entire dataset. All interview data that related to the GLC were coded.
Searching for themes	All significant patterns in the data were noted, and initial table of second-order codes and quotes created. Throughout this and subsequent stages, findings were reviewed for coherence and credibility by AI, and the raw data were regularly referred to.
Reviewing themes	From the initial table of significant second-order codes and discussions with AI, candidate themes were identified. These were then refined by repeatedly referring back to data and codes and by creating a detailed thematic map. Candidate themes were examined to establish whether they were coherent, externally heterogeneous and had explanatory power.
Defining and naming themes	Through further discussions, a more parsimonious list of themes were created. These were refined through peer debriefing and verification with LM.
Producing the paper	The paper was drafted and feedback obtained from all authors.

Dementia care mapping data, collected using the Behaviour Category Code (BCC) and ME frameworks were input into a purpose-built Excel template created by the Bradford Dementia Group. This spreadsheet was used to calculate the number of time frames and percentage of time that an individual spent in each of the BCC and ME values. Well- or ill-being scores were calculated for each person and for each group session using this tool. DCM data were recorded during eight sessions between November 2019 and March 2020. During these observations, seven people living with dementia were observed. In total, seven group sheets were created (one per session) and an additional 22 individual sheets (one per person per session). The unstructured observation field notes were thematically analysed and synthesised with the DCM descriptive analysis to provide a comprehensive account of the experiences of the GLC for participants across the sessions.

## Findings

This section presents the findings from the three phases of data collection undertaken within the evaluation.

### Before GLC: qualitative interviews

#### Well-being through gardening

Participants articulated their general love of gardening and how they enjoyed being outdoors.

FCP02: *I do like being out in the fresh air and out in the garden*.

When PLWD01 was asked by his care partner if he liked being outside in the garden, he replied “yes”. Although this was a short answer, this is of significance as PLWD01 had limited verbal communication.

A particularly poignant response from PLWD02 in relation to gardening was: Love it, love it…That’s my life… my gardening started from (*home*) I was always planting things and doing things…… and I like biology and botany.

When speaking about PLWD04, CP04 articulated the importance of the hub garden in providing PLWD04 with an outdoor space where there was the opportunity to engage in gardening.

I know (*name*) loves gardening and we haven’t got access to the big garden she used to have, when I saw it, I asked if she’d like to come…… She’s got green fingers.

Participants provided further insight into their love of gardening. Relaxation and the therapeutic benefit of gardening was important:

CP01: *I garden to release stress, if I feel a little bit stressed I'll just go out, even if it's raining I'll just nip out for a few minutes*.

It was also apparent that participants enjoyed watching the plants grow and that this gave them a sense of satisfaction and feeling of purpose.

CP01: *It's just being able to do something and actually to see the products afterwards. So like, for instance, sustainable gardening……it's a sense of achievement as well*.

The garden provides a space that is theirs, as part of the group. A fundamental part of why the garden appeals to the attendees is the sense of achievement that can be derived from gardening, whether simply watching plants grow, eating what the garden produces or giving it away. The garden is not only seen as a workspace but it is also a space to relax, which accommodates both people living with dementia and their carers.

#### Social connectedness

Participants voiced how gardening provided them with a social environment where they could connect with other people. It was evident from the data that strong social connections between members of the groups had been formed. Participants articulated the importance of sustaining and building upon these relationships through the GLC.

CP02: *I think it’s strengthened a lot of friendships, I think that’s the most important thing……I think because it’s so friendly and relaxed……there’s no pressure*.

Friendships with like-minded people in similar situations, which are based on a mutual purpose, enabled individuals to work together in ways that brought enjoyment and satisfaction. These friendships were a key motivator for current and former care partners attending the GLC.

People living with dementia reported that gardening provided them with the opportunity of connecting socially and engaging in new activities.

PLWD02: *Although I’ve got dementia, there are things I will go out of my way to do… something which is satisfying me as opposed to just watching the TV or things like that*.

PLWD02’s care partner expressed that gardening activities promoted ability and inclusivity for people living with dementia:

CP02: *it’s more appropriate to the people… that it’s emphasising ability rather than disability and that’s really important*.

A participant living with dementia illustrated the importance of gardening in providing them with a connection with others and a sense of purpose. After establishing that PLWD04 enjoyed the GLC, they were asked why. They responded:

PLWD04: *The first thing is helping people*

Former care partners highlighted that through a communal gardening group, they felt they could share their experiences and empathise with other members of the group in relation to dementia.

FCP02: *I do think that having experience of dementia sometimes, you know, if somebody says something, I can empathise with people*.

The physical space was important in promoting feelings of well-being. However, the importance of the GLC transcends the physical space and what is on offer socially can be equally important. The GLC offers a trusted social environment where members feel supported and can fulfil a need to support others.

#### Increasing knowledge

Participants reported that a motivation for them to participate within the GLC was to improve their knowledge of gardening.

CP04: *You’ll always learn more because somebody’s got some information how they do something, so there’s always that changing of information*.

It was also highlighted by a participant that they wanted to attend the GLC to learn more about dementia.

CP03: *when I got talking to the group, I wanted to know their experiences of how they handled their own experience, which helped me then to handle mine*.

This theme highlights that an important aspect of having and using the garden space is the opportunity to learn, both by sharing experiences and gaining practical knowledge. Such learning does not come from the organisers of the GLC but from the participants themselves. The varying levels of expertise create an environment where group members can learn from each other and further enhance their sense of social connection.

#### Active participation

The fourth theme to emerge was the desire to engage in a range of activities. While the GLC was formed around the garden, it was not all about gardening. The interviewees discussed their creative interests and activities they would like to spend more time doing as the GLC progresses.

FCP03: *I’d like to see some drama, I’d like to see creative writing, I’d like to see some more of the artistic side*.

Another suggestion was for more knowledge-based activities.

CP03: *I think more general knowledge things I think, to get the mind working, ‘cause dementia is about the mind……more general knowledge, quizzes, you know?*

This theme highlights the participants desire to engage in a range of activities. The GLC was seen as an opportunity to try something new or to develop an existing skill.

### During GLC: DCM data

Of the 11 GLCs that took place, eight were observed using the DCM framework as three of the sessions required increased facilitation by the researcher. Observations of eight people living with dementia were recorded, and each individual was observed at 5-minute intervals for the duration of each GLC session. Using the enhancer and detractor framework, it is possible to evaluate whether (or not) the psychological needs of ‘comfort’, ‘identity’, ‘attachment’, ‘occupation’ and ‘inclusion’ are met.

#### Mood and engagement scores (see Figure 1)

The findings show that participants spent the majority of their time engaged and experiencing good overall levels of well-being. At no point during mapping was ill-being recorded.

**Figure 1.**
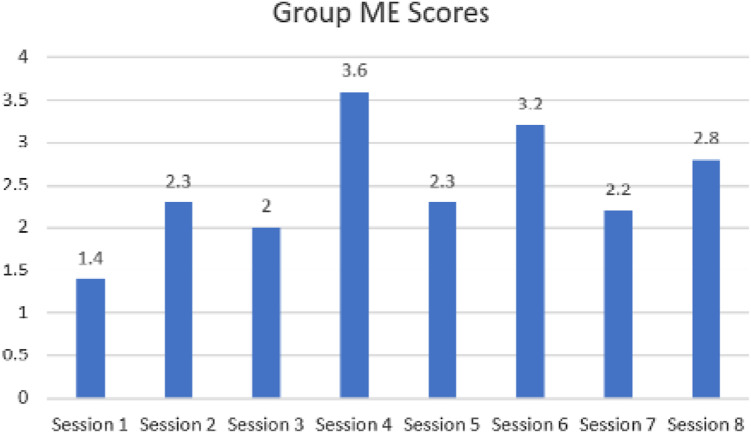
Group mood and engagement scores.

The mean ME score was +2.5 indicating a good and sustained overall level of mood and engagement across the groups. Session 1 had the lowest group ME score at 1.4 and session 4 had the highest group ME score at 3.6. During session 1, members from the University Estates team came to discuss how the purpose-built garden area might be developed and expanded, and how estates can facilitate any changes that the GLC members would like to enact. The estates team demonstrated compassion and patience, which the GLC members clearly appreciated (*PE 4. Respect – Treating the participant as a valued member of society and recognising their experience and age*)*.* While those who came actively and passionately participated, the two people living with dementia who attended this session (PLWD01 and 03) are both verbally impaired and were therefore limited in their verbal abilities to contribute to the discussion taking place. This, in consequence, resulted in lower ME scores being observed during session 1.

Session 4 was attended by 11 participants, 4 of which were people living with dementia. Session 4 of the GLC involved two separate indoor activities, one following the other. The first was as an employee of a large dementia care organisation who shared his expertise in accessible technologies for people living with dementia. The knowledge that was shared through practical demonstrations of technology was very well received, and the facilitator was experienced working with people with dementia (*PE 14. Recognition – Meeting the participant in his or her own uniqueness, bringing an open and unprejudiced attitude*)*.* The second activity was facilitated by one of the group friends who was well known to most group members. The facilitator was also experienced in dementia care, and group members were overtly happy with the baubles they produced (*PE 11. Facilitation – Assessing the level of support required and providing it*)*.* The two people living with dementia that attended session 4 were PLWD02 and PLWD05 who are both very engaged individuals, coupled with the combination of an interactive activity, enabled the highest mood and engagement scores across all the sessions. This suggests that having two simultaneous activities facilitated by experienced staff within the same session provided GLC members an opportunity to enhance well-being.

### After GLC: follow-up qualitative interviews

Five themes emerged from the follow-up interviews. Theme 5, the impact of COVID-19, emerged during the interviews and is relevant to the interpretation of the findings. There were six follow-up interviews, two interviews involving somebody living with dementia and a care partner.

#### What is a good life?

Generally, there was a sense that living a good life is about involvement and active participation in social life, particularly with friends and family. For example:

FCP02: *Well a fulfilling life, one that has meaning, I think having friends, having family, […] connecting with people. Good health is important, […] really being able, in a way, to do what you want*.

However, interestingly coming from interviewees living with dementia and current care partners, living a good life was described in mechanistic rather than social terms. For example, PLWD02 suggested that to him living a good life involved ‘*carrying on breathing*’ and that he is ‘*not doing as many [things] as [he] was doing before*’*,* which he described as being down to ‘*partly being an old fart*’ and ‘*partly not being able to do things because they*’*re not available*’. However, PLWD02 responded positively to CP02, when she stated:

CP02: *it is about being engaged in something that has purpose PLWD02 – Yes*.

CP03 (who was interviewed with PLWD03) also responded in a mixed way:

CP03: *Good life, well, not having any illnesses if you like. Having no pressures of life, you know. The good qualities of life, holidays… Family life, you know*.

And when asked if dementia has impacted their perspective on a ‘good life’, they replied:

CP03: *you’ve actually not got a good life any more as such, you know. I mean you’ve got to make the best of what you’ve actually got and in my opinion that’s not good*.

This distinction between former care partners and those currently being directly affected by dementia is interesting. However, as will be discussed in more detail below, both dyads, CP02 and PWLD02 and CP03 and PWLD03, suggest that the GLC has a positive impact on their lives and well-being. For example, when CP02 describes how the facilitator of one of the sessions got PLWD02 involved, thereby making the activity available:

CP02: *I was amazed because I’d tried to get [name of person living with dementia] involved in something like that for ages. [name of facilitator] got it*.

CP03 reflects this sentiment when they say:

CP03: *it’s a big impact on our good life if you like, you know, although it’s for a short time, it’s still a good impact, you know*.

Going back to thinking of the characteristics of a ‘good life’ as being active participation with friends and family, it is clear in the interviewees reflected these features in their descriptions of the GLC:

CP03: *The GLC… it’s important to PLWD03 and it’s important to myself because it is a little bit of a release on your normal day. The garden or we do the…the painting, or if we do the crafts, you know. Whatever it may be, it’s…it’s a big impact on our good life if you like*.

From the above quotations, it is clear that what constitutes a good life is complex and not the same for everyone. For some, a good life may be living unimpeded by illness or age-related concerns. Or ensuring a supportive social circle. There appears to be an interplay here between internal and external factors that impact on the notion of a good life. However, the GLC appears to offer some of these external factors constituting a good life.

#### Active participation

The attendees reflected positively on the autonomy afforded by the GLC, both in terms of the number of different things on offer and also the lack of prescribed ways of conducting oneself during sessions.

FCP03: *I think that's one of the good things about the club, that you do get an opportunity to do different things*.

Although participants were able to approach the GLC sessions in the way they wanted, this does not mean that there were not also excellent planned provisions. The interviewees spoke at length about how much they enjoyed the range of activities on offer.

FCP03: *the staff really did give it some thought and tried to bring in activities that we would enjoy*.

In response to a question asking what he thinks about the activities on offer, CP03 said:

CP03: [The activities on offer] *are very, very good. I think that’s the most important thing because we are so blessed to have an outside area, and we’re blessed again to have an indoor area and we can combine the two*.

There are several instances where participants report on the impact certain activities have on people living with dementias’ well-being:

FCP03: *I also noticed that, for instance, PLWD02 who quite often gets a bit distressed sometimes, I noticed we were doing one of the art activities and he became really quite engrossed in what he was doing and was concentrating on it*.

In the joint interview with PLWD02 and CP02, CP02 said:

CP02: [name of facilitator] *got you* [referring to PLWD02] *engaged in something that you really did get hold of […] That was amazing and your concentration was great. The garden has always been great. You enjoy the garden. You like pottering in the garden*. PLWD02 *Oh, yes, but not when it’s raining*.

The interviewees also reported that the GLC caters to a broader audience, and other places they had attended typically catered only for the elderly or treated people like they are unable.

CP02: *I mean, it was something…the GLC […] actually the thing that I’d been looking for, for several years. We’ve tried various things locally in our area, and PLWD02 would go a couple of times and then he’d walk out and he’d say, I’m never going back there again. Just because they were either too patronising or really uninteresting. It [the GLC] assumes people are willing, able and up for it*.

The hub space used during the GLC is not simply a place the participants come to, but it is also a place they feel ownership over. Ownership appears to be important for the members of the GLC. It is not simply that they belong, but that they are part of the decision-making processes that go into creating the GLC (and the hub). As a result, they feel both responsible for and proud of the hub.

CP02: *It’s like coming home. […] So people come into the hub, everybody knows each other, […]. No allowances are made […] It’s our space*.

#### Social connectedness and peer support

Another key theme within the interviews is the peer support experienced within GLC. The outcome of the supportive environment created by the GLC is that members are happy to seek support when feeling low and are happy to provide support when others need it.

CP02: *Yes, but I know that other people are also looking out, we’re all looking out for each other and that’s really special, actually. There aren’t many places where we can go like that*.

A key aspect of this peer support was the laughter and humour, which is the result of the relaxed atmosphere created by the environment and the sessions.

FCP05: *And of course, you'd have tea and a biscuit, or a piece of cake, and a laugh and a joke, and it was just time to relax, and forget all your worries. You'd leave them at the door at the Hub, and pick them up again on the way out*.

The group is made up of people living with dementia, carers and former carers. The findings clearly reveal that the GLC offers support specifically for current carers from past carers. For example, CP03 explains how he has and PLWD03 have benefitted from interactions with more experienced GLC members:

CP03: *I’ve certainly learned a lot… Because I was, like I said, at the very beginning when I was struggling with PLWD03’s condition, I didn’t understand it, I didn’t know at all, until it was one of the associate members wrote something, an article, in one of the magazines and I picked it up and I read it and it just changed my whole view on the way that I looked after PLWD03*.

The benefit of the GLC for carers was often mentioned within by care partners.

CP02: *I notice a difference in PLWD02 doing something that he enjoys doing that he looks forward to… and that makes my life easier undoubtedly*.

FCP05: *I think the carers like coming to the Hub. Because there is a lot for people with dementia when you look round, but there's still very little for the carers*. *[…] It's only a few places that the carers and the people with dementia can come and be together*.

From the quotes above, it is clear that an important part of the GLC is the supportive environment.

#### What could be improved?

In the follow-up interviews, participants were asked whether anything could be improved. Two themes emerged in response to this answer. One relates to the provisions provided by the GLC and the other to the number and variety of attendees.

Music was something two participants suggested they would like more of:

FCP06: *I think, I mean I know there’s a music club… but I do think music is the best thing*.

A recurrent theme in the follow-up interviews was the desire to get more people attending groups like the GLC, particularly those living with dementia.

CP03: *Well…nothing to do with the hub as such, what I would like to see is more people with dementia or Alzheimer’s coming in. There’s a lot of ex-carers there, which are absolutely great and that’s where you need the information. But, we need more people coming in*.

The members of the GLC really enjoy music, and the Institute also runs two music cafés . In addition, a music session had been planned (cancelled due to COVID-19) as part of the GLC. Recruiting new members to the GLC is an ongoing part of the work of the Institute.

#### Impact of COVID-19

Like almost everything at the time of writing, the GLC has been impacted by COVID-19. What these comments at follow-up reveal is how important the group is for those who use it, but also that there was an understanding of why it was not possible for the group to continue at the moment.

CP02: *Well, we’ve really missed it, let’s say that. That lack of social contact, lack of meeting with friends has made a massive impact really and …it has really brought home to us how isolating dementia can be for both parties, care giver and the person living with the diagnosis*.

FCP05: *Because I mean, even if we only had four people with dementia in, you imagine trying to make that a meter plus [distance], and that, it's just not big enough. And then you’ve got staff wandering about... It's an absolute nightmare*.

COVID-19 has the potential to impact on the well-being and mental health of everyone, but it is especially true of people living with dementia. Social distancing and lockdown has the potential to make people living with dementia feel isolated, and this will clearly impact on the idea of a good life, which, as shown above, involves being part of a supportive social group.

## Discussion

The findings identify four key themes. The first was that participants considered having active participation in social life to be a key aspect of living a good life. The second was that the way the GLC was set up and delivered gave the participants ownership of the GLC; they felt able to shape the club and contribute. The third was the importance of social connectedness and peer support to the well-being of both people living with dementia and care partners. A key way in which this was expressed was former care partners supporting current care partners. Fourth, positive mood and well-being was directly experienced through gardening.

To frame the discussion of the follow-up interviews, the participants were asked how they would define a ‘good life’. There was an interesting distinction between those currently being affected by dementia and former care partners. Former care partners focussed on friends and family and the importance of belonging to different social groups, while current care partners and those living with dementia discussed the mechanical aspects of life (e.g. carrying on breathing) and the medical aspects of illness. As interviews progressed, those with this more health-driven conception also discussed the importance of friendship and a shared purpose. However, everyone who was interviewed said that the GLC had a positive impact on their ability to live a good life. A dominant aspect of living a good life was self-determination; a desire to live unimpeded by either illness or by other people. People want the option to make their own decisions, to take part in activities as they choose and to be responsible for something that goes beyond any individual session. Another important aspect is to be a member of a group of friends, who offer support, advice and a source of joy.

One of the key findings is the extent participants reported feeling a sense of ownership of the GLC, meaning that they felt able to actively contribute, make choices about what they did and shape the club. There is substantial evidence that a sense of control and empowerment contributes to positive well-being (and the converse that a loss of control contributes to ill-being) (Diener & Biswas-Diener, 2005; Forgeard et al., 2011). A sense of purpose, achievement and significance are all mentioned as key to positive relationships and an enriching environment (Nolan et al., 2006). Within this study, the importance of being able to engage alongside others in activities that were personally relevant, enjoyable and meaningful was clearly linked with a sense of identity and well-being (Van Gennip et al., 2016).

Dementia support groups and dementia cafes can provide coping strategies and enjoyable/occupying activities and also offer social support, which is often associated with subjective well-being (Dow et al., 2011; Fukui et al., 2019; Greenwood et al., 2017). In addition, gardening has been found to facilitate an increased engagement for people living with dementia (Lu et al., 2020). These findings were reflected within this study, with both care partners and people living with dementia describing how they enjoyed activities and peer support offered. The analysis of the DCM data demonstrates that individuals experienced high levels of positive mood and sustained engagement when engaged in the GLC overall. More interactive and enabling sessions demonstrated higher mood and engagement scores.

Care partners and former care partners spoke about the ways the peer support networks had developed further into friendships beyond the group, extending the person’s social support system, which was also found by other similar cafes (Teahan et al., 2020). Many of the participants had become close friends, and all of them indicated that they found the hub a relaxing place where they felt comfortable. It was clear that participants take pleasure from helping each other as well as engaging in the activities.

The GLC offers members a way to promote social reserve (Wray, 2020). In that the GLC empowers individuals by offering a range of activities and allowing them to choose how they use them. This freedom reflects a positive attitude towards people living with dementia because it highlights autonomy, which in turn increases the social credibility of the individual. An opportunity to choose a course of action has the potential to limit self-stigma by promoting self-determination.

As well as advantages conferred by the synthesis of multiple data types, there are limits to the study. One limit is the reduced number of people who were able or willing to participate in follow-up interviews. There were six interviews at follow-up, and of these, two were paired interviews. The COVID-19 pandemic directly and indirectly affected participation at follow-up; however, it is hard to know to what extent the former care partners who declined to take part were influenced by COVID-19.

## Conclusions and recommendations

The GLC offers participants the opportunity to be part of a group who care for and support each other. However, the group is not simply a social group as most participants are included in the joint enterprise of creating and maintaining the garden. Those who are not as invested in the garden seem to be invested in the group itself and are keen to have input on the types of activities that form the different sessions. Within the sessions themselves, autonomy is highly valued. Individuals can choose how little or how much they engage with an activity.

The combination of in-session autonomy, in a group consisting of friends, acts as key ingredients in creating a group that is relaxed, full of humour and highly valued by its members.

The findings from this evaluation of the GLC have led us to the following recommendations that others who wish to use a café model may find useful to consider:

1. **To maximise participant well-being, groups should be set up to enable participants to actively contribute and shape the activities of the group.** Even something as simple as offering choice in activities was identified as beneficial and enhancing well-being.
2. **Groups offer a way to promote interaction and participation.** Opportunities to interact and participate with people experiencing life in similar ways were found to be an important part of everyday life to enhance participation, communication and mood.

## References

[bibr1-1471301221998897] Bamford C. Lamont S. Eccles M. Robinson L. May C. Bond J. (2004). Disclosing a diagnosis of dementia: A systematic review. International Journal of Geriatric Psychiatry, 19(2), 151-169. DOI:10.1002/gps.1050. 14758581

[bibr2-1471301221998897] Bradford Dementia Group . (2005). DCM 8 user’s manual. Bradford Dementia Group.

[bibr3-1471301221998897] Braun V. Clarke V. (2006). Using thematic analysis in psychology. Qualitative Research in Psychology, 3(2), 77-101. DOI:10.1191/1478088706qp063oa.

[bibr4-1471301221998897] Brooker D. J. Surr C. (2006). Dementia care mapping (DCM): Initial validation of DCM 8 in UK field trials. International Journal of Geriatric Psychiatry: A Journal of the Psychiatry of Late Life and Allied Sciences, 21(11), 1018-1025. DOI:10.1002/gps.1600. 16955431

[bibr5-1471301221998897] Department of Health & Social Care . (2020). Prime Minister's challenge on dementia 2020. Gov.uk.

[bibr6-1471301221998897] Dewing J. (2007). Participatory research: A method for process consent with persons who have dementia. Dementia, 6(1), 11-25. DOI:10.1177/1471301207075625.

[bibr7-1471301221998897] Diener E. Biswas-Diener R. (2005). Psychological empowerment and subjective well-being. In D. Narayan (Ed.), Measuring empowerment: Cross-disciplinary perspectives (pp. 125-140). The World Bank. DOI:10.1037/e597202012-007.

[bibr8-1471301221998897] Doka K. J. (2010). Grief, multiple loss and dementia. Bereavement Care, 29(3), 15-20. DOI:10.1080/02682621.2010.522374.

[bibr9-1471301221998897] Dow B. Haralambous B. Hempton C. Hunt S. Calleja D. (2011). Evaluation of Alzheimer's Australia Vic memory lane cafés. International Psychogeriatrics, 23(2), 246-255. DOI:10.1017/S1041610210001560. 20670462

[bibr10-1471301221998897] Forgeard M. J. Haigh E. A. Beck A. T. Davidson R. J. Henn F. A. Maier S. F. Mayberg H. S. Seligman M. E. (2011). Beyond depression: Toward a process‐based approach to research, diagnosis, and treatment. Clinical Psychology: Science and Practice, 18(4), 275-299. DOI:10.1111/j.1468-2850.2011.01259.x. 22509072 PMC3325764

[bibr11-1471301221998897] Fukui C. Fujisaki-Sueda-Sakai M. Yokouchi N. Sumikawa Y. Horinuki F. Baba A. Suto M. Okada H. Ogino R. Park H. (2019). Needs of persons with dementia and their family caregivers in dementia cafés. Aging clinical and experimental research, 31(12), 1807-1816. DOI:10.1007/s40520-019-01129-2. 30694511

[bibr12-1471301221998897] Gonzalez M. T. Kirkevold M. (2014). Benefits of sensory garden and horticultural activities in dementia care: A modified scoping review. Journal of Clinical Nursing, 23(19-20), 2698-2715. DOI:10.1111/jocn.12388. 24128125

[bibr13-1471301221998897] Gov.UK . (2020). Coronavirus (COVID-19): Guidance and support. Gov.uk.

[bibr14-1471301221998897] Greenwood N. Smith R. Akhtar F. Richardson A. (2017). A qualitative study of carers’ experiences of dementia cafés: A place to feel supported and be yourself. BMC Geriatrics, 17(1), 1-9. DOI:10.1186/s12877-017-0559-4. 28743253 PMC5527402

[bibr15-1471301221998897] Hall J. Mitchell G. Webber C. Johnson K. (2018). Effect of horticultural therapy on wellbeing among dementia day care programme participants: A mixed-methods study (Innovative Practice). Dementia, 17(5), 611-620. DOI:10.1177/1471301216643847. 27072371

[bibr16-1471301221998897] Jarrott S. E. Gigliotti C. M. (2010). Comparing responses to horticultural-based and traditional activities in dementia care programs. American Journal of Alzheimer's Disease & Other Dementias®, 25(8), 657-665. DOI:10.1177/1533317510385810. PMC10845608 21131672

[bibr17-1471301221998897] Lu L.-C. Lan S.-H. Hsieh Y.-P. Yen Y.-Y. Chen J.-C. Lan S.-J. (2020). Horticultural therapy in patients with dementia: A systematic review and meta-analysis. American Journal of Alzheimer's Disease & Other Dementias®, 35, 1533317519883498. DOI:10.1177/1533317519883498. PMC10623907 31690084

[bibr18-1471301221998897] Moniz-Cook E. Vernooij-Dassen M. Woods B. Orrell M. Interdem N. (2011). Psychosocial interventions in dementia care research: The INTERDEM manifesto. Aging & Mental Health, 15(3), 283-290. DOI:10.1080/13607863.2010.543665. 21491215

[bibr19-1471301221998897] Nolan M. Brown J. Davies S. Nolan J. Keady J. (2006). The senses framework: Improving care for older people through a relationship-centred approach. Getting research into practice (GRiP) report No 2.

[bibr20-1471301221998897] Noone S. Innes A. Kelly F. Mayers A. (2017). ‘The nourishing soil of the soul’: The role of horticultural therapy in promoting well-being in community-dwelling people with dementia. Dementia, 16(7), 897-910. DOI:10.1177/1471301215623889. 26701960

[bibr21-1471301221998897] Noone S. Jenkins N. (2018). Digging for dementia: Exploring the experience of community gardening from the perspectives of people with dementia. Aging & Mental Health, 22(7), 881-888. DOI:10.1080/13607863.2017.1393793. 29068703

[bibr22-1471301221998897] Social Care Institute for Excellence . (2015). What the research says: Dementia - support following diagnosis. https://www.scie.org.uk/dementia/after-diagnosis/support/research.asp

[bibr23-1471301221998897] Teahan Á. Fitzgerald C. O'Shea E. (2020). Family carers’ perspectives of the Alzheimer Café in Ireland. HRB Open Research, 3, 18. DOI:10.12688/hrbopenres.13040.1. 32518892 PMC7243203

[bibr24-1471301221998897] Van Gennip I. Pasman H. Oosterveld-Vlug M. Willems D. Onwuteaka-Philipsen B. (2016). How dementia affects personal dignity: A qualitative study on the perspective of individuals with mild to moderate Dementia. The Journals of Gerontology. Series B, Psychological Sciences and Social Sciences, 71(3), 491. DOI:10.1093/geronb/gbu137. 25271308

[bibr25-1471301221998897] Wray A. (2020). The dynamics of dementia communication. Oxford University Press.

